# Addressing Socioeconomic Barriers to Timely Antenatal Care Among Working Women in Indonesia

**DOI:** 10.34172/jrhs.12735

**Published:** 2026-08-15

**Authors:** Agung Dwi Laksono

**Affiliations:** ^1^Research Center for Public Health and Nutrition, National Research and Innovation Agency, Republic of Indonesia, Jakarta, Indonesia; ^2^Persakmi Institute, Surabaya, Indonesia

**Keywords:** Prenatal care, Socioeconomic factors, Employed women, Maternal health, Indonesia, Health equity

## Abstract

**Introduction:**

Female workers in Indonesia represent a particularly vulnerable population, as economic pressures frequently compel them to assume dual roles in income generation and family caregiving. Accordingly, this study examined the influence of socioeconomic factors on the delayed initiation of antenatal care (ANC) among this group of women.

**Study Design:**

A cross-sectional study.

**Methods:**

Utilizing secondary data from the 2023 Indonesian Health Survey, this secondary analysis included 30,173 women of reproductive age who had given birth within the previous five years. The primary outcome was delayed ANC initiation, with socioeconomic status as the primary exposure variable. Residence, age, marital status, education, health insurance, and parity were also included as control variables. SPSS 26 and binary logistic regression were utilized for the statistical analysis.

**Results:**

Nearly 18.0% of working women experienced delayed initiation of ANC. Socioeconomic status significantly influenced ANC timeliness. Compared with the wealthiest group, the likelihood of delayed ANC initiation progressively increased across lower wealth categories: the poorest (95% confidence interval [CI]: 2.814–2.855), poorer (95% CI: 1.624–1.647), middle (95% CI: 1.542–1.562), and richer (95% CI: 1.367–1.384). The narrow CIs reflect the large sample size and the survey data’s high statistical precision.

**Conclusion:**

The findings demonstrate a clear socioeconomic gap in timely maternal healthcare. It was revealed that low-income working women need specific interventions to overcome financial and structural barriers to ANC. Moreover, they require more financial protection and occupational flexibility to receive ANC and improve maternal health in Indonesia.

## Introduction

 This secondary analysis occurs because maternal mortality is a global issue, particularly in low-income and middle-income countries, including Indonesia.^1^ It reflects improvements in healthcare accessibility and quality and is a key indicator of national health.^2^ By 2030, the Sustainable Development Goals (SDGs) aim to reduce maternal mortality to 70 per 100,000 live births worldwide, thereby preventing any nation from exceeding 140 per 100,000 live births.^3^ Indonesia’s maternal mortality ratio (MMR) was 346 per 100,000 live births in the 2010 Population Census. Nonetheless, it dropped to 189 in the 2020 Long Form Census.^4^ Despite a 45% reduction in maternal mortality over the past decade, Indonesia faces difficulty achieving the SDG target within five years.

 Indonesia’s 2020 MMR was 1.3 times the Southeast Asia regional average, the fourth-highest behind Cambodia (218), East Timor (204), and Myanmar (179) per 100,000 live births.^5^ According to the report of the World Health Organization (WHO), the MMR of Southeast Asia dropped from 231 to 74 deaths per 100,000 live births from 2000 to 2020, while that of Indonesia reduced from 299 to 189.^5^ Indonesia’s 42.8% decline in MMR (15.2%) was lower than the region’s 58% drop. It is possible to prevent most maternal deaths by using antenatal care (ANC) visits and following the recommended periodicity.^2^

 Pregnant women need trained healthcare practitioners’ ANC. Obstetric hemorrhage, anemia, eclampsia, and preterm birth must be detected early.^6^ ANC promotes delivery care and educates mothers and children about health.^7^ In addition, ANC services are cost-effective and can decrease MMR by 20% in low-income countries, according to a study.^8^ According to WHO guidelines for safe motherhood, the Indonesian Ministry of Health recommends 6 ANC visits: 2 in the first trimester, 1 in the second, and 3 in the third.^9^ Beyond frequency, ANC also affects the quality and timing of visits, which are crucial to maternal health.^3^ A Ugandan study reported that ANC visits reduce maternal and infant mortality by helping women prepare for birth, recognize pregnancy risks, and respond properly.^10^

 Moreover, quick ANC services can prevent pregnancy issues and preexisting disease from worsening before they become life-threatening.^11^ However, delaying or ignoring ANC raises the risk of low birth weight, premature birth, hypertension, and *postpartum hemorrhage*.^12^ Furthermore, a late ANC start may affect results more than visit frequency.^13^

 Socioeconomic disparities in ANC usage are well known; most research assumes women as a homogeneous population and ignores work status as a moderator. Women workers face additional obstacles beyond poverty. Due to household wealth, demanding work schedules, fear of income loss, and a lack of workplace support, economically active women may delay care. These restrictions affect Indonesia, where most women work informally without social protection. The mechanisms by which socioeconomic status influences the timely initiation of ANC among working women are unknown. This gap must be addressed to inform the health system and labor organizations’ maternal health equality policies.^10,14^ Therefore, this study links socioeconomic status to delayed ANC, enabling more equitable and responsive policies to ensure all women receive timely and high-quality prenatal care.^8^ More precisely, this study aims to investigate socioeconomic determinants and delayed ANC beginning among Indonesian working women.

## Methods

###  Data Source and Study Design

 The research utilized secondary data from the 2023 Indonesian Health Survey. The Ministry of Health supervises national health affairs. The research employed multistage, stratified random sampling to integrate home and individual-instrument interviews. The survey was conducted from May to July 2023, with a response rate of 91.49% among participants ^15^.

 The study used secondary data from the 2023 Indonesian Health Survey, a nationally standardized survey performed by Indonesia’s Ministry of Health. The survey instruments were developed centrally, pilot-tested, and implemented using standardized procedures across all provinces. Content validity was ensured through alignment with internationally accepted maternal health indicators and WHO guidelines, particularly for ANC measurement. Additionally, construct validity has been supported by extensive prior use of these indicators in peer-reviewed studies and national reports. Reliability was also maintained through rigorous enumerator training, standardized questionnaires, multistage stratified sampling, and continuous field supervision. Data quality control procedures included real-time consistency checks and post-survey validation conducted by the survey authority, ensuring reliable and comparable measurements across regions.

 The research included Indonesian working women who had delivered five years earlier. In this study, working women were defined according to the 2023 Indonesian Health Survey classification. A woman was considered employed if she reported engaging in any income-generating activity at the time of the survey, including formal or informal employment, self-employment, and unpaid family work. This definition captures a broad spectrum of labor participation, reflecting the reality of women’s employment in Indonesia, where informal and family-based work constitute a substantial share of female economic activity. However, women who reported not working at the time of the survey were excluded from the analysis. The inclusion criteria for this study were women of reproductive age who were working women, had given birth within the 5 years preceding the 2023 Indonesian Health Survey, and had complete information on ANC initiation and socioeconomic status. On the other hand, the exclusion criteria included women who were not working at the time of the survey, women who had not experienced a live birth within the specified five-year period, and respondents with missing or incomplete data on key study variables, such as the timing of the first ANC visit, wealth index, or selected control variables. Applying these criteria resulted in a final analytic sample of 30,173 female workers.

###  Setting

 The study was nationally conducted in Indonesia.

###  Outcome Variable

 The delayed initiation of ANC visits among pregnant women was the outcome variable. The first ANC visit was considered delayed if it occurred beyond 16 weeks of pregnancy. If the pregnancy was older than 4 months, the first ANC visit in the past 4 months was designated as “1”. If not, it was regarded as early initiation if it was scheduled within four months and indicated by the code “0”.

###  Exposure Variable

 The research employed socioeconomic status as an exposure variable. This study evaluated a household’s value based on its possessions. In addition, housing was assessed based on access to potable water, restroom facilities, first-floor materials, and the quantity and type of amenities, including televisions, bicycles, and automobiles. The figure was derived by principal component analysis; the average score of each family member categorized 20% of the population into five wealth groups nationally. The survey classified individuals by wealth into the poorest, poorer, middle, more affluent, and richest groups.^16^

 Socioeconomic status was measured using the standard wealth index derived from household assets and housing characteristics. While this index is widely used and validated for population-level analyses, it represents long-term household economic status rather than short-term or individual-level economic conditions. Accordingly, it may not fully capture the financial realities faced by working women, particularly variations in cash flow, income stability, job security, or control over personal earnings. It should be noted that these factors are especially relevant for employed women, whose access to ANC may depend on daily wages, informal employment arrangements, or unpredictable income. Consequently, the wealth index should be interpreted as a proxy for structural economic position rather than a direct measure of financial liquidity or employment-related economic vulnerability.

###  Control Variables

 This study investigated six control factors, such as type of residence, age group, education level, marital status, insurance coverage, and parity. The study included age groups ≤ 19, 20–24, 25–29, 30–34, 35–39, 40–44, and ≥ 45. Further, the intended women had different levels of education: elementary, junior, senior high school, and college. Moreover, their marital status included married, divorced, or widowed.

 Furthermore, the study categorized health insurance ownership into four groups: uninsured, government-run insurance, private-run insurance, and mixed (encompassing both government-run and private-run insurance). Parity is the number of live births a woman has ever had, comprising primiparous (≤ 1), multiparous (2–4), and grand multiparous (> 4).

###  Data Analysis

 The chi-squared test was employed to analyze two variables in the initial segment. Moreover, the collinearity test was used to establish the absence of a significant relationship among the independent variables in the second segment of the investigation. Additionally, the entry approach was applied for the final binary logistic regression analysis. Adjusted odds ratios (aORs) from the survey were presented alongside 95% confidence intervals (CIs). IBM SPSS (version 26) was utilized for the statistical analysis.

## Results

 Among 30,173 Indonesian female workers who had delivered within the previous 5 years, 18.0% delayed the initiation of ANC. Figure 1 shows a strong positive correlation between the proportion of delayed ANC and the poverty prevalence across provinces (R^2^ = 0.666). Based on the results, provinces with higher proportions of poor women (e.g., Papua and East Nusa Tenggara) had higher rates of delayed ANC initiation. In contrast, wealthier provinces (e.g., Jakarta and Bali) displayed lower delays.

**Figure 1:**
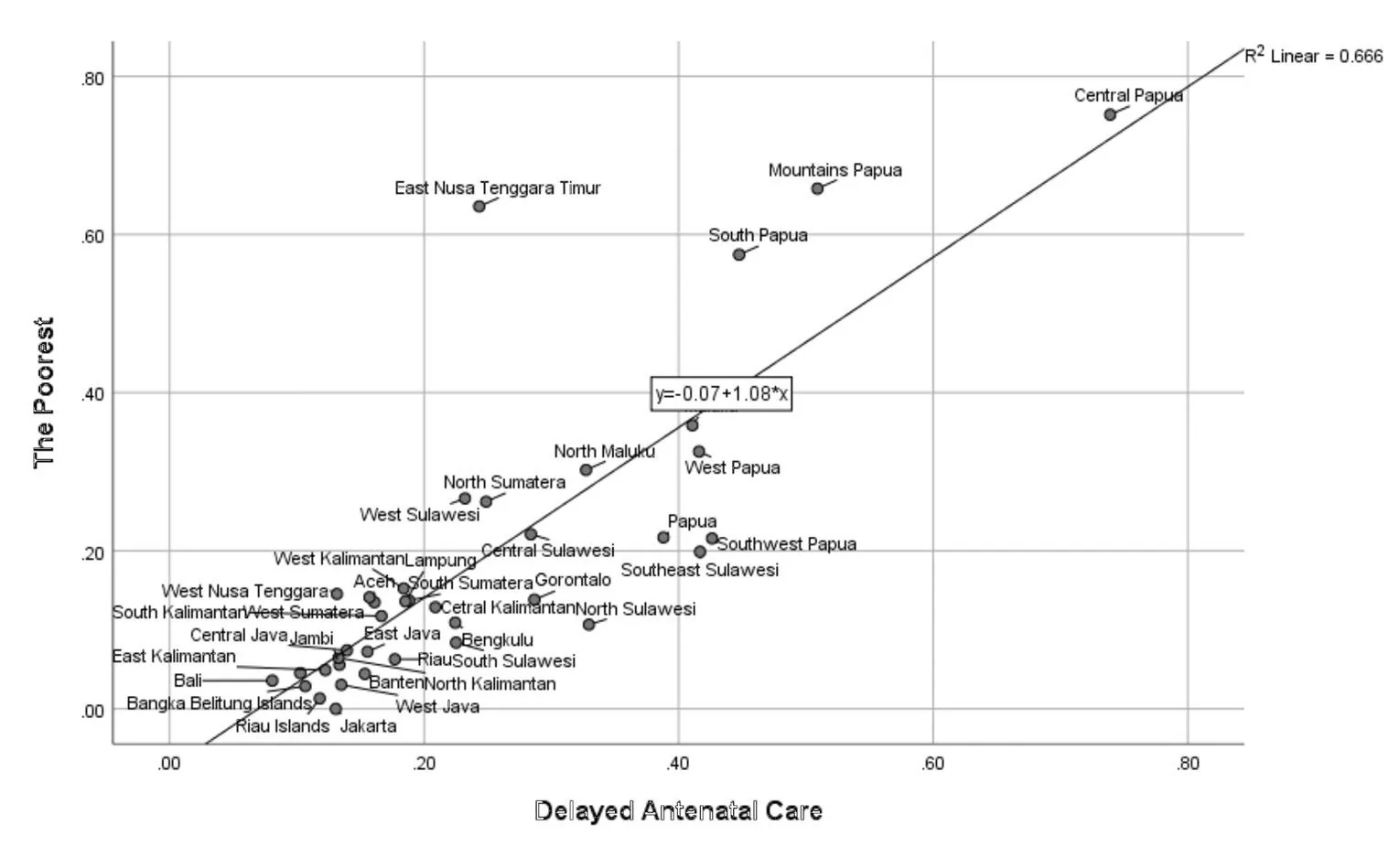
Scatter Plot of the Proportion of Delayed Antenatal Care Initiation and the Poorest Proportion by Province Among Indonesian Female Workers

 Moreover, Table 1 presents a descriptive analysis revealing a clear socioeconomic gradient. The proportion of delayed ANC initiation decreased consistently from the poorest to the richest groups, from 34.6% among the poorest to 10.7% among the richest. In addition, delays were more common among those living in rural areas, women aged 25–34 years, and those with lower educational attainment. Likewise, most female workers across all wealth groups were married and covered by government-run health insurance. All variables were significant at *P* < 0.001.

**Table 1:** The Results of Descriptive Analysis (N = 30,173)

**Variables**	**Socioeconomic Status**					***P*** **-Value**
	**Poorest,** **n=5,517**	**Poorer,** **n=4,781**	**Middle,** **n=5,309**	**Richer,** **n=6,188**	**Richest,** **n=8,378**	
Delayed antenatal care initiation						0.001
No Yes	19,733	23,867	24,410	25,345	26,944	
	10,440	6,306	5,763	4,828	3,229	
Residence						0.001
Urban Rural	5,733	10,621	14,453	18,979	23,776	
	24,440	19,552	15,720	11,194	6,397	
Age group (year)						0.001
< 19 20-24 25-29 30-34 35-39 40-44 ≥ 45	573	181	272	151	0	
	5,944	4,526	3,410	2,836	1,207	
	7,483	7,996	8,177	8,931	7,996	
	7,121	8,117	8,931	9,112	10,410	
	5,310	5,733	6,125	6,035	7,181	
	2,746	2,836	2,595	2,625	2,776	
	996	784	664	483	603	
Marital status						0.001
Married Divorced/widowed	29,057	28,695	28,966	29,117	29,600	
	1,116	1,478	1,207	1,056	573	
Education level						0.001
No education Primary school Junior high school Senior high school College	1,931	784	302	181	121	
	11,134	7,724	5,823	3,349	724	
	7,060	8,479	7,362	5,793	2,142	
	8,237	10,410	12,160	13,216	9,897	
	1,810	2,776	4,556	7,634	17,289	
Health insurance						
Uninsured Government-run Private-run Mixed	9,595	7,272	7,332	5,884	3,168	
	20,306	22,630	22,268	23,565	25,225	
	181	181	453	634	1,297	
	91	91	121	91	483	
Parity						0.001
Primiparous Multiparous Grand multiparous	9,082	9,535	9,022	9,565	9,414	
	18,918	19,431	20,367	20,035	20,156	
	2,172	1,237	784	573	603	

 The second phase of the investigation involved assessing collinearity. Based on the test results, independent variables demonstrated a weak or nonexistent correlation. Every factor possessed a tolerance value of 0.10 or higher. Further, the variance inflation factor for each variable was below 10.00, implying that the regression model is devoid of multicollinearity.

Table 2 presents the output of multivariate analysis. Binary logistic regression confirmed that socioeconomic status was a strong predictor of delayed ANC initiation. Compared with the wealthiest group, the odds of delayed ANC were significantly higher among the poorest (aOR: 2.835, 95% CI: 2.814–2.855), poorer (aOR: 1.636, 95% CI: 1.624–1.647), middle (aOR: 1.552, 95% CI: 1.542–1.562), and richer (aOR: 1.375, 95% CI: 1.367–1.384) groups. Additionally, rural residents were more likely to delay ANC than urban residents (aOR: 1.120, 95% CI: 1.116–1.125).

**Table 2:** The Results of the Collinearity Test

**Independent Variables**	**Collinearity Statistics**	
	**Tolerance**	**VIF**
Socioeconomic status	0.664	10.506
Residence	0.813	10.230
Age group	0.735	10.360
Marital status	0.994	10.006
Education level	0.720	10.389
Health insurance	0.934	10.070
Parity	0.730	10.370

*Note*. VIF: Variance inflation factor; Dependent variable: Delayed antenatal care.

 Based on the findings (Table 3), women with lower education levels, divorced or widowed women, and those with higher parity were more likely to delay ANC initiation. In contrast, having any form of health insurance (government, private, or mixed) could significantly reduce the likelihood of delay compared with being uninsured.

**Table 3:** The Association of Antenatal Care and Various Variables Using Binary Logistic Regression (N = 30,173)

**Variables**	**Adjusted OR (95% CI)**	***P*** **-Value**
Socioeconomic status		
Riches Richer Middle Poorer Poorest	Ref.	
	1.375 (1.367, 1.384)	0.001
	1.552 (1.542, 1.562)	0.001
	1.636 (1.624, 1.647)	0.001
	2.835 (2.814, 2.855)	0.001
Residential area		
Urba Rural	Ref.	
	1.120 (1.116, 1.125)	0.001
Age groups (year)		
≤ 19 20–24 25–29 30–34 35–39 40–44 ≥ 45	Ref.	
	0.268 (0.263, 0.273)	0.001
	0.215 (0.210, 0.219)	0.001
	0.192 (0.188, 0.195)	0.001
	0.222 (0.218, 0.227)	0.001
	0.233 (0.228, 0.238)	0.001
	0.352 (0.344, 0.360)	0.001
Marital status		
Married Divorced/widowed	Ref.	
	1.304 (1.292, 1.317)	0.001
Educational level		
College Senior high school Junior high school Primary school No education	Ref.	
	1.180 (1.173, 1.187)	0.001
	1.215 (1.207, 1.223)	0.001
	1.462 (1.451, 1.472)	0.001
	2.420 (2.389, 2.453)	0.001
Insurance status		
Uninsured Government-run Private-run Mixed	Ref.	
	0.765 (0.762, 0.769)	0.001
	0.763 (0.751, 0.775)	0.001
	0.483 (0.468, 0.498)	0.001
Parity		
Primiparous Multiparous Grand multiparous	Ref.	
	1.302 (1.295, 1.309)	0.001
	2.513 (2.487, 2.540)	0.001

*Note*. OR: Odds ratio; CI: Confidence interval.

## Discussion

 The study revealed that socioeconomic status was associated with delayed initiation of ANC among Indonesian female workers. Furthermore, the possibility of delaying ANC initiation was higher when the women were poorer, highlighting a significant disparity in access to essential maternal health services. Women from lower socioeconomic backgrounds frequently encounter numerous obstacles, including restricted financial resources, insufficient transportation, rigid work schedules, and a lack of awareness regarding the significance of early ANC, which might postpone their initial engagement with healthcare professionals throughout pregnancy. This delay may also hinder the early identification of serious health issues for both the mother and fetus, ultimately jeopardizing maternal and neonatal outcomes.^17,18^

 Although the findings demonstrate a socioeconomic gradient in delayed ANC initiation, these patterns among working women are best understood through the combined effects of financial constraints and work-related time poverty. Pregnancy does not reduce economic necessity for low-income female workers. Instead, it often intensifies the need to maintain continuous employment, particularly in informal or low-wage jobs where income loss from even short absences can threaten household stability. Thus, this financial pressure, combined with limited workplace flexibility, makes it difficult for women to attend ANC during standard clinic hours without sacrificing wages or job security. As a result, ANC is frequently postponed until pregnancy becomes more visible or symptoms arise, rather than being initiated early for preventive purposes.^17–20^

 Time poverty further exacerbates these challenges. Working women frequently balance paid labor with unpaid domestic responsibilities, leaving limited discretionary time for seeking healthcare. Even when services are nominally affordable or insured, the opportunity costs of time spent traveling to facilities, waiting for services, and recovering from clinic visits can outweigh the perceived benefits of early ANC. These structural constraints help explain why insurance coverage demonstrates a strong protective effect, as it reduces direct costs and may facilitate access to higher-level facilities with more predictable service delivery. Overall, these dynamics suggest that delayed ANC among working women is not primarily a matter of individual preference or awareness, but a rational response to constrained choices shaped by employment conditions and economic precarity.^17–20^

 Regarding residence type, female workers in rural areas were more likely to delay the initiation of ANC than those in urban areas, reflecting enduring spatial differences in access to maternal health services. Women in rural areas frequently encounter restricted access to healthcare facilities, extended travel lengths, inadequate transportation infrastructure, and a deficiency of skilled healthcare professionals. These obstacles may significantly hinder timely ANC visits, particularly for employed women who must balance professional obligations with domestic commitments. This indicates the need for improved healthcare infrastructure, expanded outreach services, and tailored interventions in rural areas to ensure that all pregnant women, regardless of their location, receive timely and adequate prenatal care.^21–23^

 The study revealed that age, education, and marital status (three maternal characteristics) are associated with delayed initiation of ANC among Indonesian female workers. The association between maternal age, education, and marital status and delayed initiation of ANC among Indonesian female workers reflects the interaction between individual characteristics and employment-related pressures. Younger working women may lack both experience and adequate knowledge of the importance of early ANC, and these gaps are often compounded by limited workplace bargaining power, fear of income loss, or concerns about job security when requesting time off for health visits. Similarly, women with lower educational attainment may face additional challenges in navigating health information and administrative procedures, particularly in formal work settings that require documentation or prior leave scheduling, which can discourage early ANC utilization.^24–28^

 In addition, marital status intersects with employment conditions to shape health-seeking behavior. Divorced or widowed working women may experience reduced social and financial support, increasing their dependence on continuous employment and limiting their flexibility to prioritize antenatal care. In some work environments, unmarried or previously married pregnant women may also encounter stigma, which can intensify reluctance to disclose pregnancy early and delay ANC initiation. In general, these findings demonstrate that maternal characteristics influence ANC timing through their interaction with workplace constraints and social expectations, representing the need for employment-sensitive maternal health interventions that address both knowledge gaps and structural barriers within work settings.^24–28^

 Across health insurance status groups, all types of health insurance were less likely to delay ANC initiation than the uninsured, revealing the crucial role of financial coverage in ensuring prompt access to maternity care. Health insurance alleviates the economic burden of medical bills, enabling female workers to access early prenatal care without the fear of substantial out-of-pocket costs. This suggests that insurance coverage enhances affordability and may help increase health awareness and utilization through associated services and outreach efforts.^29,30^ Enhancing and fortifying health insurance programs, particularly for informal and low-income workers, may be a pivotal approach to augment early ANC uptake and mitigate maternal health inequities in Indonesia.

 Furthermore, parity types were more likely to delay the initiation of ANC than primiparous women, indicating that prior birthing experience may reduce the urgency to seek early prenatal treatment. Multiparous women may consider themselves well-informed about pregnancy, leading them to undervalue the significance of appropriate ANC in subsequent pregnancies. The responsibilities of caring for several children, coupled with work obligations, may restrict their capacity to prioritize early health check-ups. This issue represents the necessity for ongoing education and support for all pregnant women, irrespective of parity, to stress that each pregnancy presents distinct risks and necessitates prompt and suitable care. A study in Addis Ababa, Ethiopia, reported that excessive parity was associated with a substantial negative correlation with timely ANC initiation.^31^

 The most vital point of this research was the examination of large volumes of data to provide insights at the national level in Indonesia. Conversely, the survey limited the variables analyzed to those relevant to the secondary data. This study had several limitations. First, the analysis relied on secondary data from the 2023 Indonesian Health Survey. While this dataset offers strong national representativeness and standardized measurement, it restricts the range of variables that can be examined. In particular, key workplace-related factors (e.g., employment type, working hours, workplace flexibility, employer support for maternal health, and maternity leave arrangements) were not captured in this study. The absence of these variables limits the ability to fully explain how working conditions directly influence the timing of ANC initiation among employed women.

 In addition, the survey did not include indicators of women’s decision-making autonomy or household power dynamics, which are known to affect maternal health-seeking behavior. As a result, the observed associations between socioeconomic status, insurance coverage, and delayed ANC initiation may be partially confounded by unmeasured social and workplace factors, suggesting that the findings should be interpreted as reflecting broad structural and economic determinants rather than specific causal pathways. Therefore, future studies should incorporate primary data collection or mixed-methods approaches that explicitly measure workplace conditions and women’s autonomy to generate more nuanced and policy-relevant evidence.^32,33^

**Highlights**

- Nearly one in five working women in Indonesia delayed antenatal care (ANC) initiation.
- Lower socioeconomic status remarkably increased the likelihood of delayed ANC.
- Health insurance consistently reduced the risk of delayed ANC among working women.
- Financial pressure and limited work flexibility remain key barriers to timely maternal care.

## Conclusion

 This study demonstrated a transparent socioeconomic gradient in the timely initiation of ANC among working women in Indonesia, with low-income women facing substantially higher risks of delay. Among all determinants examined in this study, health insurance coverage revealed the strongest and most consistent protective effect against delayed ANC initiation. Therefore, the most immediate and actionable policy priority is to expand and strengthen health insurance coverage for working women, particularly those in low-income and informal employment sectors. Ensuring automatic enrollment, reducing administrative barriers, and improving benefit awareness can significantly accelerate early ANC uptake and narrow maternal health inequities.

 Beyond financial protection, complementary strategies, such as flexible service hours and outreach in rural areas, remain essential. Nonetheless, insurance expansion should be positioned as the central policy lever given its demonstrated impact. Future research should move beyond household and individual characteristics in order to explicitly capture workplace-related factors, including employment type, job security, working hours, maternity leave, and employer support for maternal health. Integrating these variables will be essential to designing more responsive labor and health policies that effectively support timely ANC for working women.

## Acknowledgments

 The author would like to express his gratitude to the Ministry of Health of Indonesia for consenting to assess the 2023 Indonesian Health Survey data.

## Artificial Intelligence Use Disclosure

 In this manuscript, AI-based tools were used solely to support language editing and improve grammar and clarity of expression. The author is responsible for all scientific ideas, data analysis and interpretation, conclusions, and the generation of original scientific content. Moreover, he takes full responsibility for the accuracy, integrity, and originality of the manuscript and has carefully reviewed and approved the final version prior to submission.

## Competing Interests

 The authors declare they have no competing interests.

## Ethical Approval

 The 2023 Indonesian Health Survey was authorized by the National Ethics Committee (No. LB.02.01/I/KE/L/287/2023). In addition, written informed consent was obtained for the survey, and participants or their guardians provided signed consent to account for the voluntary and private nature of the data collection method.

## Funding

 This study was self-funded by the authors and received no external financial support from any funding organization.
